# ICARES: a real-time automated detection tool for clusters of infectious diseases in the Netherlands

**DOI:** 10.1186/s12879-017-2300-5

**Published:** 2017-03-09

**Authors:** Geert H. Groeneveld, Anton Dalhuijsen, Chakib Kara-Zaïtri, Bob Hamilton, Margot W. de Waal, Jaap T. van Dissel, Jim E. van Steenbergen

**Affiliations:** 1 Department of Internal Medicine and Infectious Diseases, Leiden University Medical Center, P.O. box 9600, 2300 RC Leiden, The Netherlands; 2 Unit for Infectious Disease Control, Public Health Service Hollands Midden, Leiden, The Netherlands; 3 Faculty of Engineering and Informatics, University of Bradford, Bradford, UK; 4 inFact, Shipley, UK; 5 Department of Public Health and Primary Care, Leiden University Medical Center, Leiden, The Netherlands; 6 Centre for Infectious Disease Control, National Institute for Public Health and the Environment (Rijksinstituut voor Volksgezondheid en Milieu, RIVM), Bilthoven, The Netherlands; 7 Department of Infectious Diseases, Leiden University Medical Center, Leiden, The Netherlands

**Keywords:** Cluster detection, Respiratory tract infection, Meningoencephalitis, Hepatitis, Real-time, Automated

## Abstract

**Background:**

Clusters of infectious diseases are frequently detected late. Real-time, detailed information about an evolving cluster and possible associated conditions is essential for local policy makers, travelers planning to visit the area, and the local population. This is currently illustrated in the Zika virus outbreak.

**Methods:**

In the Netherlands, ICARES (Integrated Crisis Alert and Response System) has been developed and tested on three syndromes as an automated, real-time tool for early detection of clusters of infectious diseases. From local general practices, General Practice Out-of-Hours services and a hospital, the numbers of routinely used syndrome codes for three piloted tracts i.e., respiratory tract infection, hepatitis and encephalitis/meningitis, are sent on a daily basis to a central unit of infectious disease control. Historic data combined with information about patients’ syndromes, age cohort, gender and postal code area have been used to detect clusters of cases.

**Results:**

During the first 2 years, two out of eight alerts appeared to be a real cluster. The first was part of the seasonal increase in Enterovirus encephalitis and the second was a remarkably long lasting influenza season with high peak incidence.

**Conclusions:**

This tool is believed to be the first flexible automated, real-time cluster detection system for infectious diseases, based on physician information from both general practitioners and hospitals. ICARES is able to detect and follow small regional clusters in real time and can handle any diseases entity that is regularly registered by first line physicians. Its value will be improved when more health care institutions agree to link up with ICARES thus improving further the signal-to-noise ratio.

## Background

Worldwide, the number of infectious disease outbreaks is increasing [1]. Consequently, the early detection of and response to clusters of infectious diseases is becoming more important.

Past experience shows that many outbreaks of infectious diseases are detected late. For example, in the Netherlands in 1999, a point source outbreak of Legionnaire’s disease was detected 14 days after the first patient was admitted to hospital. At that time, another 70 patients had already been admitted to various hospitals throughout the Netherlands [2, 3].

There are many similar examples where retrospective analysis of data clearly indicates that clusters of infectious diseases are not detected until relatively late. This hampers the identification of the source of the outbreak, the control of the associated transmission route(s) and the identification of associated conditions.

For example, delayed detection of hemolytic uremic syndrome (HUS) and bloody diarrhea of Shiga Toxin-producing Escherichia coli outbreak in Germany in 2011 had significant and long-lasting impacts [4, 5]. The speculation about the association between the Zika virus outbreak and microcephaly gave rise to conflicting advice to women of childbearing age [6, 7].

Such delayed detections and lack of detailed insight in possible related conditions are costly in terms of the disease burden but also have impact on the social and economic aspects of the communities affected [8].

Reasons for late detection can be attributed to the non-specificity of the detection systems. For example, Google Flu Trends was developed to find a potential flu cluster as soon as possible. Critical analysis revealed that it has overestimated the number of flu cases and Google Flu Trends has discontinued to publish current estimates [9, 10].

This large amount of data noise can be overcome by medical doctors being the data source. Medical doctors define a working diagnosis at first patient contact. Such primary data yield more specific results in comparison with lay persons based systems as Google Flu Trends.

On the other hand, using disease syndromes in outbreak surveillance frequently lacks specificity and commonly refers to a broader categorisation, e.g., respiratory tract infection or gastro-enteritis. Additionally, General Practitioners (GPs) do not, for instance, regularly request microbiological testing for these syndromes. This can easily result in a missed opportunity to successfully identify a possible cluster that could represent the first sign of a much larger potential outbreak.

To overcome this information gap, the Dutch Public Health Law (Wet Publieke Gezondheid), based on the International Health Regulations (IHR) [11], obliges medical doctors to report unusual clusters of infectious diseases with possible serious public health consequences. The criteria for reporting under this heading are not well specified and in practice medical doctors hardly ever report such clusters. Still, individual physicians will miss clusters in overlapping physician catchment areas. This is clearly exemplified by the aforementioned examples.

The gaps in surveillance intelligence described above highlight the urgent need for a surveillance tool to capture and analyse regional clusters of infectious diseases. This tool should ideally be automated, real-time and based on diseases identified by medical doctors without adding to the administrative burden of medical professionals [12, 13]. This will prompt public health professionals to investigate further when certain upper limits of incidence for a given syndrome have been reached. Detailed information about the extent of an outbreak will help public health authorities to inform and advice the involved population adequately. Our case study addresses this gap specifically.

## Methods

From 1 October 2013 to 1 October 2015, a pilot ICARES (Integrated Crisis Alert and Response System) case study was conducted in the Leiden-The Hague region in the western part of the Netherlands. This area has approximately 1.25 million inhabitants, six hospitals, eight GP Out-of-Office-Hours services and 380 individual GP practices.

This study was approved by the Medical Ethical Committee of the Leiden University Medical Center on 18 April 2012. The aim of the case study was to design, develop and test an automated surveillance tool capable of providing early signals of potential clusters that could escalate into outbreaks. The complete spectrum of front-line health care organisations contributed to this case study and included General Practices, Out-of-Hours General Practitioner services, and one hospital (emergency department, ward and intensive care unit admissions and outpatient department consultations). For the hospital, DBC/DOT (Diagnose Behandel Code Op weg naar Transparantie) codes were used to map to the corresponding syndrome. Hospital physicians routinely enter codes during the first evaluation of a patient. These DBC/DOT codes are developed for hospitals to reimburse the costs of patient care at health care insurers and represent the patient’s diagnosis.

Diagnostic information from General Practitioner (GP) patient records is obtained using the International Classification of Primary Care (ICPC) [14], according to the guidelines of the Dutch College of General Practitioners [15]. Nowadays, both in daily practice and during out-of-office hours, GPs routinely enter these codes in the electronic patient file at first patient presentation.

Any disease entity that is routinely coded and entered in the patient record can be selected. In this case study, we focused on respiratory tract infection, infectious hepatitis and meningoencephalitis.

Trigger diagnostic codes (Table 1) are collected and sent to ICARES every 24 h, yielding a near real-time snapshot of what is happening in the community and its burden on health care institutions. Together with these diagnostic codes, the minimal data set (MDS) of patient sex, age range, the four digits of the postal district (i.e., not the full postal code), identification of the participating health care facility and date of consultation are captured for transmission to ICARES. For reasons of data confidentiality, privacy and security, no specific patient identifiable information is collected from the GP systems. With hospital data, an encrypted patient identification number is added, with only the principal investigator at the hospital being able to decrypt these codes. This practice ensures that the minimal data set does not contain patient identifiable information.

**Table 1:** Trigger diagnostic codes

DBC/DOT code (Hospital)ª	Representing syndrome/diagnosis
Respiratory tract infection	
INT401	Pneumonia
INT402	Interstitial pneumonia
INT409	Other respiratory tract infections
LON1401	Pneumonia
LON1405	Acute (trachea)bronchitis
KIN3104	Upper respiratory tract infection
KIN3202^b^	Asthma/bronchial hyperreactivity
KIN3207	Laryngotracheobronchitis
KIN3208	Lower respiratory tract infection
KIN3210	RSV bronchiolitis
Infectious hepatitis	
INT463	Viral hepatitis (not B or C)
INT944	Hepatitis B or C
MDL701	Hepatitis
MDL705	Hepatitis B or C with antiviral therapy
MDL718	Acute liver failure
KIN3312	Hepatitis
Meningitis/encephalitis	
INT441	Meningitis/encephalitis/brain abscess
NEU0101	Bacterial Meningitis
NEU0102	Non-bacterial meningitis
NEU0111	Encephalitis
KIN3511	Meningitis/encephalitis
**ICPC (General Practice)**	**Representing syndrome/diagnosis**
Respiratory tract infection	
R74	Acute upper respiratory tract infection
R77	Acute laryngitis/tracheitis
R78	Acute bronchitis/bronchiolitis
R80	Influenza
R81	Pneumonia
Infectious hepatitis	
D13	Icterus
D72	Infectious hepatitis
Meningitis/encephalitis	
N70	Poliomyelitis/(entero)viral infection CNS
N71	Meningitis/encephalitis

^a^DBC/DOT codes from internal medicine, pulmonology, pediatrics, neurology and gastroenterology are used

^b^This code is only used in children under the age of 5 since asthma/bronchial hyperreactivity, at this age, is most often triggered by a respiratory tract infection

In order to obtain calculation baselines for the data analysis, historic data from the various participating organisations were collected and analysed. This case study benefited from 1 year’s data from GPs, including GP Out-of-Office-Hours services, as well as 8 years of hospital data. This yielded means and standard deviations for various codes.

A secure web-based decision support tool was developed for the purpose of this study by inFact Ltd. and was named ICARES (Integrated Crisis Alert and Response System). The software tool receives the MDS from the various participating organisations every night. Special web services have been written to interface, in a non-intrusive way, with the disparate electronic patient records. ICARES then maps all the diagnostic codes received onto the corresponding three sets of syndromes mentioned, and presents the analysed data in an easy to understand dashboard with a risk dial for each disease to the local unit of infectious disease control.

ICARES aggregates the actual data harvested and compares these values with those for the nearest current time window historically. Calculations in ICARES are currently performed using this Cumulative sum (CUSUM) method for a moving seven-day period [16]. To calculate the equivalent historic period, the previous seven-day period is taken into consideration, adjusted for holidays.

The above information is synthesised in a risk dial with traffic light colors immediately recognisable as green to signify a normal setting, orange when a warning threshold has been reached corresponding to an incident ratio between 0.75 and 1.40 and red for an incident ratio of more than 1.40. The rates can only be calculated for the GP population since it is only in the GP practices that the number of patients, the denominator, is known. For hospital and Out-of-Hours General Practitioner services, colors are determined by rates of the 7-day numbers observed divided by the historic 7-day numbers. Thresholds are the same as those for incident ratio.

These colors on the dashboard provide a crude indication of current numbers versus historic numbers. If colors turn red, more profound investigation is warranted to define whether further action is needed. These action limits are visualized in the graphs and defined by three standard deviations above average.

Should the ICARES action limit be exceeded, i.e., indicating that a possible cluster is detected for that given institution, the local unit of infectious disease control will use this as a trigger for further investigation. After assessment of geographic information and raw data, they will consult the treating physicians to find out more about the specific diagnosis and patient characteristics of the possible cluster. Up-to-date information continues to be available on the dashboard in order to follow the cluster as it evolves over time. If a specific, microbiologically confirmed diagnosis is not available at the time when the trigger appears, diagnostic protocols for possible outbreaks have been put in place to deal with this. Parts of these protocols are adapted from current national guidelines [17].

The dashboard is an easy to use quick scan for possible clusters. If colors and numbers are within normal range, no further action is necessary and the dashboard can be reopened the next day. This visual quick scan of the dashboard is done daily by the local unit for infectious disease control in the Leiden-the Hague area and by the research team and takes less than one minute.

All alerts will be evaluated whether it have been real clusters or not. Reasons for false positive alerts will be documented as well as the use of additional, public health care initiated, diagnostic tests.

## Results

ICARES, the automated, real-time tool for the detection of clusters of infectious diseases has been tested on three disease entities since October 2013: respiratory tract infection, infectious hepatitis and meningoencephalitis.

After a run-in period of 3 months, the project started with one teaching hospital participating (catchment area approximately 200,000 inhabitants) and four GP practices with 33,117 patients [18]. During the first 24 months, four Out-of-Hours General Practitioner services (catchment area approximately 500,000 inhabitants) and ten more GP practices joined, contributing to a total number of 78,924 GP patients [19, 20].

GP coverage in the complete Leiden-The Hague study area was 6%. Since most of the health care facilities were located in the Leiden part of the study area, GP coverage in the Leiden region was 11%. Coverage of Out-of-Hours GP services in the Leiden region was 67%, hospital coverage was 27%.

On a daily basis, the local unit of infectious disease control and the research team checked the risk dials on the ICARES dashboard.

In the first 2 years of ICARES, eight signals of possible clusters were detected. Two of these alerts appeared to be a real cluster. Characteristics are outlined in Table 2.

**Table 2:** Alerts during the first 2 years of ICARES

Alert	Syndrome (Health care institution)	Additional public health diagnostics	True cluster	Comment
1	Respiratory tract infection (GP)	No	No	Different causative agents and coding imperfections
2	Infectious hepatitis (GP)	Yes	No	Non-infectious hepatitis
3	Meningoencephalitis (Hospital)	No	Yes	Enterovirus encephalitis
4	Meningoencephalitis (Hospital)	No	No	Two unrelated cases of Listeria in Katwijk
5	Infectious hepatitis (GP)	No	No	Coding imperfections
6	Respiratory tract infection (Hospital and GP)	No	Yes	Long lasting influenza season with high peak incidence
7	Meningoencephalitis (Hospital)	No	No	Coding imperfections/double coding
8	Meningoencephalitis (GP)	No	No	Non-acute illness

**Fig. 1:**
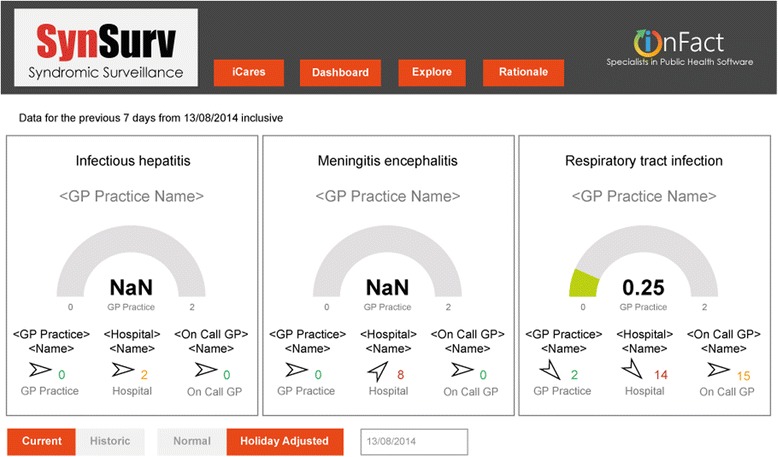
Dashboard on 13 August 2014 during meningoencephalitis outbreak. Dial numbers are incident ratios: the ratio between the observed previous 7 days incident rate with the equivalent historic incident rate. Rates are calculated as the numbers of incidents per 100,000 as based upon the GP practice’s population data. The dial color is set as *green* for an incident ratio of less than 0.75, orange for between 0.75 and 1.40 and *red* for greater than 1.40. Dials are limited to GP practices as these are the only ones where population data is available. *Colored numbers* are absolute incident counts for the last 7 days for a given institution. The institution that is displayed, is the one with the largest incident ratio. This is the ratio between observed and historic using rate values if available, otherwise absolute counts. The color is determined in a similar manner to the dial color. Trend *arrows* are determined from the ratio between the current week’s (previous 7 days) observed incident rate (or observed absolute incident count if rate not available) and the same value as calculated for the previous week. The trend *arrow* reflects current week versus previous week. A rising trend is shown for ratios greater than 1.1, stable for between 0.9 and 1.1, and falling for less than 0.9. NaN = Not a Number. NaN is displayed when the equivalent historic 7 day period has zero cases. A ratio would result in a divide by zero error

**Fig. 2:**
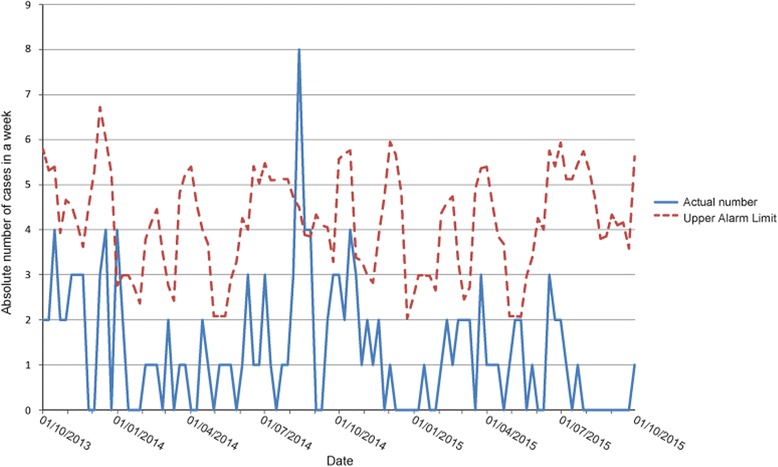
Hospital cases of meningoencephalitis 1/10/2013–1/10/2015

Alert 3 was detected from August 8 2014 onwards (Fig. 1). Eight cases of meningoencephalitis were reported within 1 week in the hospital (Figs. 1 and 2). Prompt analysis ultimately revealed that three cases with Enterovirus encephalitis belonged to the same cluster. Two of these three were household contacts. The third case was from a different four-digit postal district.

The other five notifications from the cluster of meningoencephalitis were double coded or had another cause than Enterovirus. Daily evaluation of this cluster revealed a sharp decline in incidence after 1 week.

The peak in meningoencephalitis cases occurred during the Enterovirus season, which was also detected, retrospectively, by the virologic surveillance program in the Netherlands [21].

**Fig. 3:**
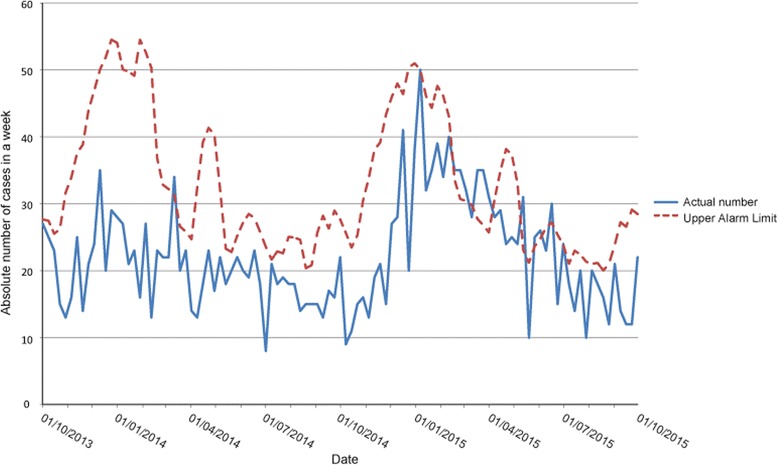
Hospital cases of respiratory tract infections 1/10/2013–1/10/2015

Alert 6 consisted of influenza cases in March-May 2015 (Fig. 3). It was part of the 2014–2015 influenza season which was remarkably long lasting and had a higher peak incidence compared to previous influenza seasons.

**Fig. 4:**
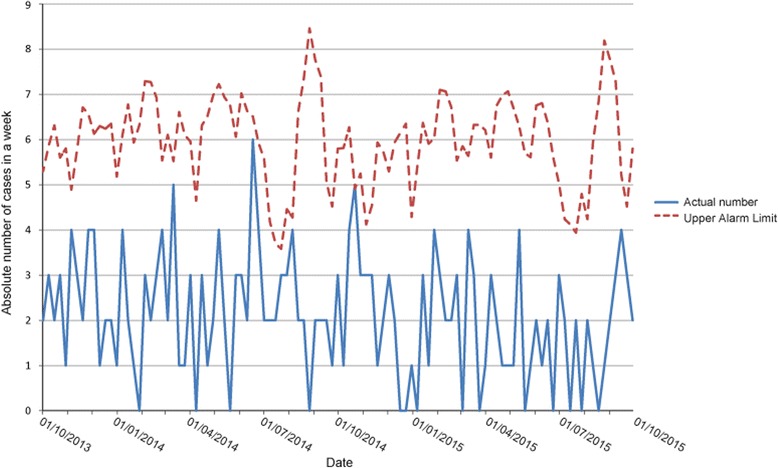
Hospital cases of hepatitis 1/10/2013–1/10/2015

Figure 4 represents hepatitis cases in the hospital. Numbers during study period did not exceed the upper alarm limit.

Two alerts were not analysed. From March 6 2014 onwards, a small peak of respiratory tract infections was detected (Fig. 3). This alert coincided with a late, minor peak in Influenza-like illness, detected by national surveillance system. It was therefore not analyzed further.

On December 26 2013, the threshold for meningoencephalitis was exceeded (Fig. 2). Discussion by the research team concluded that this could not be a real cluster, partly because of the low absolute numbers. Further evaluation was abandoned.

## Discussion

We developed and tested ICARES as an automated, real-time tool for the detection of clusters of infectious diseases. In a small pilot region, ICARES detected differences in incidence in the three groups of diseases in real time (24-h window) during the first 2 years of the project. Alert 3 and alert 6 demonstrate the ability of ICARES to detect and to monitor clusters of infectious diseases in real time.

Important strengths of ICARES are the robust diagnosis data with the minimal data set, the real-time collection and easily interpretable presentation of disease data, the historic comparison specific for each health care provider, the absence of administrative burden for medical professionals and the flexibility of the system.

Disease data should be very specific and we therefore opted in our project for definition by a medical doctor. In the Dutch health care system, doctors enter a diagnostic code in their medical record routinely. This diagnostic code most likely has a higher reliability than data used by other detection tools as Google Flu Trends and Triple S, using non-specific health indicators and proxy measures to define a syndrome [22]. In our case study, the exceedingly long lasting flu season of 2014/2015 was notified and no significant alert was generated for the mild 2013/2014 flu season. On top of that, ICARES will represent the health care consumption in possible outbreaks since all patients in ICARES did visit a medical doctor.

Another strength of ICARES is the minimal data set. Details relating to geographic mapping or age cohort are important for source detection in the early phases of a possible outbreak. The minimal data set is non-patient specific and fully respects data privacy laws. But, if required, individual hospital-patient data can be traced by the treating physician since an encrypted patient identification number can be decrypted by the principal investigator in the hospital. At GP level, the treating GP can share information by finding the cases in a possible cluster via a query in their own GP information system. Diagnostics to evaluate the cluster (and the individual patient’s illness) can be advised to treating physicians by public health care professionals. This was done during the second alert.

Daily, new data from health care providers are compared with their own historic numbers. Without significant changes in coding custom or patient population, this entails that the percentage of double coded patients or travelers would be the same in both historic group and current patients making false positive clusters for these reasons less likely.

Data acquisition and presentation on a dashboard are done daily. This contains the real-time character of ICARES enabling public health authorities to analyse clusters at an earlier stage. Other comparable systems, such as the Electronic Surveillance System for the Early Notification of Community-Based Epidemics (ESSENCE), show the difficulty in detecting an outbreak soon enough to start up control measures [23]. So far, the limited amount of small clusters detected with ICARES is insufficient to evaluate its real-time character and to determine its ability to slow the spread of infection.

As shown in the third alert with a cluster of Enterovirus encephalitis, updates on the evolution of the cluster are made available on a daily basis enabling public health care authorities to inform policy makers and public adequately.

On the other hand, when numbers of infectious diseases are not above alarm threshold, a quick scan of the dashboard is usually enough to reassure public health care authorities.

The codes used for ICARES make it possible to capture clusters of a wide range of diseases via the three selected syndromes. Even new emerging infectious diseases presenting as one of these syndromes can be detected via ICARES. To implement ICARES fully, other syndromes will be added in the future. Also, in case of newly arising possible disease associations, any other disease entity might be selected for this type of surveillance.

An important reason is that ICARES algorithm is not based on a static threshold before triggering an alert. Seasonal variations in the incidence of syndromes warrant adjusting the baseline values of syndromes. The ICARES algorithm with adjusting baseline values for seasonal variations in the incidence of syndromes, gives rise to a moving threshold for cluster detection. The pragmatic and mature SPC-based (Statistical Process Control) algorithm used in ICARES can readily be used in most generalized case studies. Various challenges arising from shortcomings of other methods have been explored by various authors [24–28]. CUSUM charts seem to adapt better to this type of analysis as they help improve the consideration of seasonal patterns as mentioned by Fricker et al. [29].

This case study has several limitations as well.

Signal-to-noise ratio was questionable during this case study with two real clusters versus six false positive alerts. Positive predictive value is therefore 0.25. Although we are not aware of any missed clusters, we cannot calculate sensitivity.

Imperfections in coding for a new patient with a non-specific syndrome may constitute reasons for low signal-to-noise ratio. This may result in false positive alerts. This is illustrated by the alert 1, 5 and 7. Other reasons for false positive alerts might be provoked by other factors contributing to a syndrome resembling an infectious disease. A sudden increase in respiratory symptoms can be attributed to a contagious viral infection but also, e.g., to a high pollen count.

The relatively small number of health care facilities and, with that, the limited regional coverage during this first 2 years of ICARES may give rise to false positive and false negative alerts.

The historic data from our GPs only cover a 1-year period and are therefore not robust. Eight-year historic hospital data might be too long as changes in care and population might make the oldest data irrelevant for upcoming cluster definition. Further work is therefore required to determine the appropriate length of history.

Currently, GP data is aggregated according to the underlying patient population data. This is not possible when considering hospitals and Out-of-Hours GP services as the exact catchment area is not known. As regional coverage broadens, assessment of this catchment area will also improve and incidence rates can be calculated for all health care facilities based on the total population in the (public health) district. As more health care facilities join the ICARES project, improved mathematical modelling to define alarm thresholds will be necessary.

Alerts are visible for public health care authorities within 24 h after the treating physician routinely enters the trigger code. General Practitioners enter the ICPC code during the first consultation, DBC/DOT codes in hospital should be entered at first patient presentation. However, DBC/DOT codes can be changed when initial diagnosis changes and whether medical doctors abide by instant coding, is unknown. This could hamper real-time detection of clusters.

ICARES is a new and unique surveillance tool in the Netherlands to detect clusters of diseases in real time. Current local detection of small clusters depends on notification by medical doctors or laboratories as is defined in the Dutch Public Health Law (Wet Publieke Gezondheid), based on the International Health Regulations (IHR) [11]. Nationwide, weekly updates of virological results are published [21] and weekly updates about patients visiting their GP with influenza-like illness are reported [30]. Automated tools for real-time detection of clusters are lacking. Systems for detection of acute hepatitis or meningoencephalitis are lacking as well.

Therefore, ICARES can improve outbreak detection in the Netherlands when used as a complement rather than a substitute for human involvement in interpreting cluster detection.

Diagnostic protocols in possible clusters have not been tested sufficiently during this project. It would be interesting to explore more disease syndromes, like food-borne diseases. This might improve its use for public health care authorities.

Further implementation of ICARES will enable cost benefit analysis. At this stage, maintenance costs are less than €10.000,- per year; daily efforts of local units of infectious disease control are minimal in case no thresholds are being exceeded. Besides time expenditure of existing staff, the development and primary piloting costs did not surpass €100,000.-.

Benefits will depend on the appearance of any clusters of infectious disease and the contribution of ICARES as a complement of surveillance tools in order to curb the outbreak.

To cite an outbreak that would have benefitted from an automated surveillance system, the current Zika epidemic in South America is an example. We could survey the illness as well as complications like microcephaly and Guillain Barre syndrome by adding diagnostic codes to ICARES.

As the project evolved, more institutions have expressed their willingness to participate. At the time of writing of this paper (22 November 2016) four hospitals, four Out-of-Hours General Practitioner services and 25 GP practices (87,380 patients) submit their consultation data daily. For GP patients, this leads to a coverage of approximately 12% in the Leiden region. There is still some way to go to improve regional coverage and robustness of data.

## Conclusions

ICARES was able to detect and to monitor local clusters of infectious diseases automatically and in real-time. Therefore it could be a complement to current surveillance tools in the Netherlands and other countries with highly digitalized health care administrations.

## Abbreviations

CUSUM: Cumulative sum
DBC/DOT: Diagnose Behandel Code Op weg naar Transparantie
GP: General Practitioner
HUS: Hemolytic uremic syndrome
ICPC: International Classification of Primary Care
IHR: International Health Regulations
MDS: Minimal data set
NaN: Not a Number
SPC: Statistical Process Control
